# IOAT: an interactive tool for statistical analysis of omics data and clinical data

**DOI:** 10.1186/s12859-021-04253-x

**Published:** 2021-06-15

**Authors:** Lanlan Wu, Fei Liu, Hongmin Cai

**Affiliations:** 1 Department of Software Engineering, South China University of Technology, Guangzhou, China; 2 Department of Computer Science and Technology, South China University of Technology, Guangzhou, China

**Keywords:** Feature selection, Cancer subtypes, Multi-omics data integration, Clinical data, Risk assessment, Multi-omic clustering, Survival analysis, Safety

## Abstract

**Background:**

With the development of high-throughput sequencing technology, a huge amount of multi-omics data has been accumulated. Although there are many software tools for statistical analysis and visual development of omics data, these tools are not suitable for private data and non-technical users. Besides, most of these tools have specialized in only one or perhaps a few data typesare, without combining clinical information. What’s more, users could not choose data processing and model selection flexibly when using these tools.

**Results:**

To help non-technical users to understand and analyze private multi-omics data and ensure data security, we developed an interactive desk tool for statistical analysis and visualization of omics and clinical data (shortly IOAT). Our mainly targets csv format data, and combines clinical data with high-dimensional multi-omics data. It also contains various operations, such as data preprocessing, feature selection, risk assessment, clustering, and survival analysis. By using this tool, users can safely and conveniently try a combination of various methods on their private multi-omics data to find a model suitable for their data, conduct risk assessment and determine their cancer subtypes. At the same time, the tool can also provide them with references to genes that are closely related to tumor staging, facilitating the development of precision oncology. We review IOAT’s main features and demonstrate its analysis capabilities on a lung from TCGA.

**Conclusions:**

IOAT is a local desktop tool, which provides a set of multi-omics data integration solutions. It can quickly perform a complete analysis of cancer genome data for subtype discovery and biomarker identification without security issues and writing any code. Thus, our tool can enable cancer biologists and biomedicine researchers to analyze their data more easily and safely. IOAT can be downloaded for free from https://github.com/WlSunshine/IOAT-software.

## Background

With the development of high-throughput sequencing technology, massive multi-omics data have been accumulated, including genomics, epigenetics, and transcriptomics. The in-depth integration and analysis of these omics data combined with clinical data can structurally observe and describe diseases (especially tumors) from multiple molecular levels, thereby achieving comprehensive molecular typing of patients, promoting the development of precision medicine, and broadening horizons in biomarker discovery [1].

Although many multi-omics data analysis tools already exist,there are still many problems. (1) Those tools have traditionally specialized in only one or perhaps a few data types. While these complex datasets generate insights individually, integrating with other-omics datasets is crucial to help researchers discover and validate findings. (2) They provide a relatively fixed calculation process, which cannot provide users with various flexible methods, including preprocessing, training models, clustering, and so on. Moreover, they cannot combine different models for users to choose, such as the UCSC Xena [2] and Firehose [3]. We also found that these tools do not completely combine multi-omics data with clinical data to carry out molecular subtype research. (3) In our research, many web tools have been developed for multi-omics data analysis, which is excellent in analyzing public data. However, uploading private data to a server beyond the user’s control poses a significant security risk. Not only that, in our test, when web-based tools upload user data (lung cancer data used by IOAT), many tools crash due to the large data set and cross-regional issues, resulting in a very poor user experience.

To address those issues, we developed an interactive tool for statistical analysis of omics and clinical data (shortly IOAT), which enables non-technical users to perform research on private high-dimensional multi-omics data without any programming burden and security risks. The tool reads data from a comma-separated value (CSV) text file, which containing multiple omics data and clinical data. Then, it can analyze multiple omics data of different integration types and flexibly perform various operations such as data preprocessing, feature selection, clustering and survival analysis. Users can select different feature selection methods for the data to find a method suitable for this type of data, and perform a risk assessment on the selected features. They can set the *K* value by self or adopt the value selected by the system to cluster the filtered features. Then, the *K* value with better survival analysis results as the subtype classification result to provide first-line doctors and scientists with specific cancer molecular subtypes reference. At the same time, the tool provides the function of survival analysis on some omics data or clinical data. In addition, the tool supports the operation of saving the current result to the specified location in each operation step.

The Firehose tool outputs the results of cancer molecular subtypes to a fixed paper template. But our IOAT is to output user data preprocessing results, feature selection models and results, risk assessment results, clustering results, survival analysis results and visualization graphs, and the overall user usage time into a report. Hence, our tool provides users with more complete data training process, which helps them better understand the data. The results of feature selection can provide them with genes closely related to tumor staging, which can be used as a reference for the connection between omics and clinical phenotypes, and help to establish a personalized cancer treatment plan. Finally, IOAT is a desktop tool that can ensure the privacy of patient data and does not require any network support. Researchers can explore their data, under data security.

## Implementation

IOAT is a standalone Windows application that has a fast fully interactive graphical user interface. It is was written in Python and R, and runs over the freely available python runtime environment, taking advantage of its strong computational engine and editable graphical outputs. IOAT is freely available for download at https://github.com/WlSunshine/IOAT-software.

We packaged the codes and compiled them into an executable file, ensuring the security of user private data. Users could use our IOAT without any installation and configuration. In the subsequent development, we plan to use two forms to analyze the functions of the software. For public data (TCGA, etc.), all functions of IOAT desktop tool are displayed to users in web form, so that users can better analyze some public data sets. For the user’s private data, users can directly choose our local desktop tool IOAT to ensure the privacy security.

**Fig. 1:**
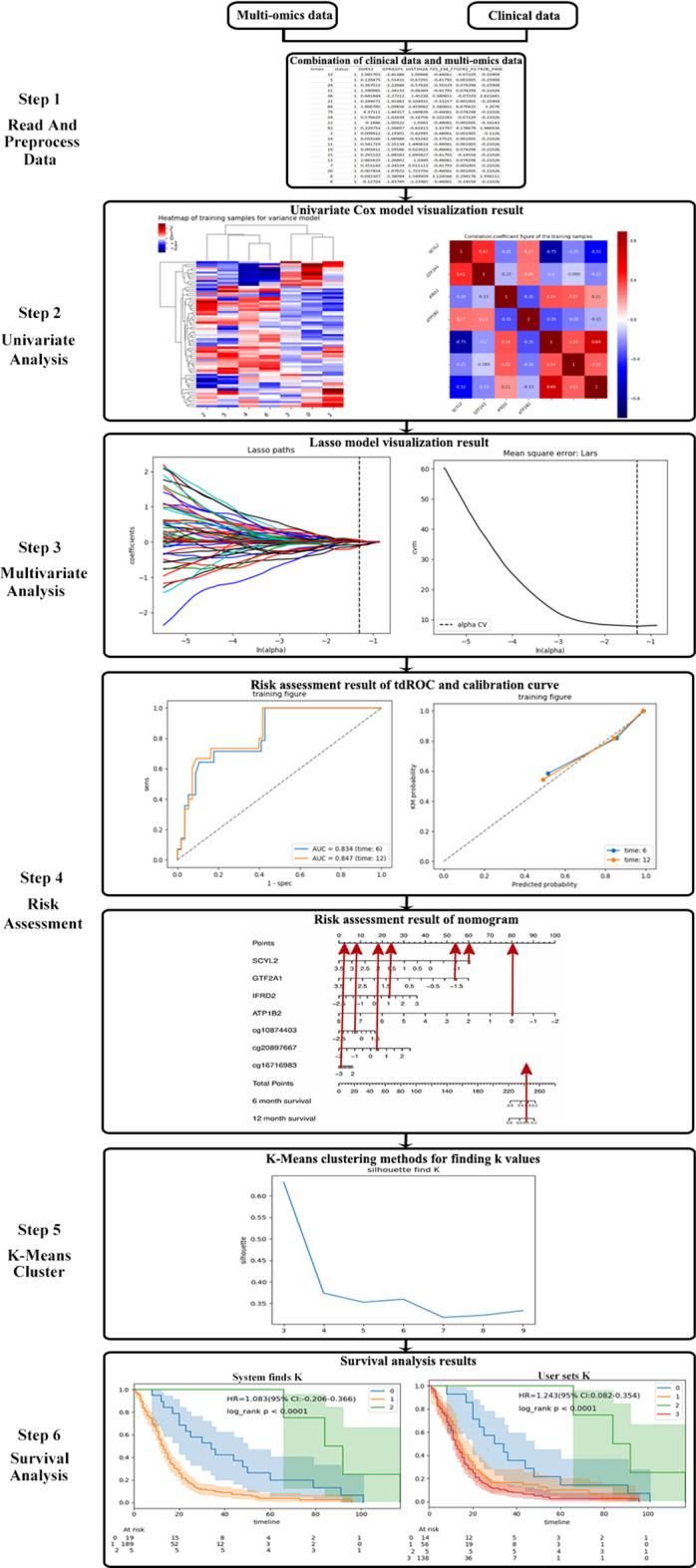
The software operation process taking master lung cancer as an examples: Step 1 read and preprocess data; Step 2 feature selection of Univiarate Cox model: Hierarchical clustering heat map and correlation coefficient figure; Step 3 feature selection of Multiviarate Lasso model: Lasso path map and Mean square error graph; Step 4 risk assessment: the selected features are used for risk assessment to predict the survival rate of patients, and the effect of the model is evaluated through td-ROC curve and c-index. Step 5 KMeans cluster: the graph between the correlation coefficient and the *K* value; Step 6 Survival analysis: chart with the system selecting the optimal *K* value and the user setting the *K* value

Compared with other tools, IOAT is very safely and conveniently to non-programmers and private data, it does not require users to have any programming foundation. It combines a variety of multi-omics data and clinical data to better study the life cycle of different cancer patients. Our IOAT identifies distinct subgroups in cancers based on different private omics data, which is performed on personal computer and would not leak data. According to the characteristics after screening, a risk assessment is carried out to predict patient survival rate. The analysis workflow of IOAT is given in Fig. 1, taking lung cancer as an example: Data are imported and preprocessed. Perform single-factor and multi-factor analysis on high-dimensional multi-omics data to reduce the feature dimension and find features that are closely related to cancer. The selected features are used for risk assessment to predict the survival rate of patients, and the effect of the model is evaluated through td-ROC curve and c-index. Perform KMeans clustering on the selected multi-omics data to obtain different molecular subtypes. Perform survival analysis according to different molecular subtypes to test whether there are significant differences between groups.

**Fig. 2:**
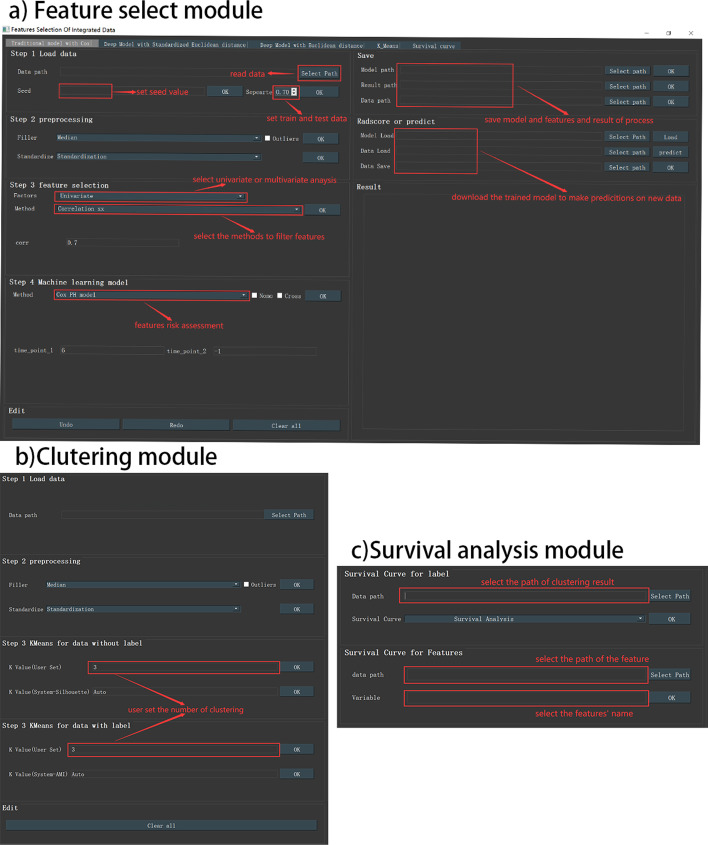
**a** The module of feature selection and risk assessment. **b** The module of clustering. **c** The module of survival

IOAT’s main screen (Fig. 2) includes several key functions.The results of feature selection and clustering analysis can be saved in the user-specified location for an unlimited number of times. The heat map, cluster node map, survival analysis map, etc. obtained from each operation can also be saved by the user in the designated location. When there is an error operation, the error in the data processing will be displayed in the ‘Result’ result column, and the user has been informed whether the operation is correct or whether the model is suitable for this type of data. After another error occurs, the user can also click the “Redo” button to make the operation go back to the previous step, and click the ‘Save’ button to save the current result and model (Fig. 2a, b). When users want to analyze new data, they can click on the ‘Clear all’ button to clear all operations and start a new exploratory research. Every operation step of the user will be recorded in the ‘Result’ column (Fig. 2b).

In this paper, we describe IOAT’s main features, including data preprocessing, feature selection, risk assessment, clustering, and survival analysis. Moreover, we take the lung cancer multi-omics data as an example, set the random seed node to 1, the segmentation data set is 7:3, the median fills in the missing values of the data, and the standardized data set. Then a variety of model combination methods are used for data preprocessing, and the effect of the model is evaluated through risk assessment and survival analysis. Finally find the most suitable method for the lung cancer data.

## Results

We now describe IOAT’s main features, organized by analysis steps (Fig. 2). The described features can be accessed using IOAT’s menus or graphical user interface.The dataset used was TCGA’s lung omics data .

### Data loading and preprocessing

At the first step, IOAT reads a comma-separated value (csv) text file to import both omics and clinical data by asking the user to click the “Select Path” button (see Fig. 1 Step 1). The user can set a random seed and a proportion to segment the training and test set data. The default proportion is set to 0.7 which means the training set takes up 70% of the whole data set.

At the second step, IOAT preprocesses the imported data by offering the following operations: (1) outlier eliminating, (2) filling missing values with either mean or median values, and (3) feature scaling with either Standardization or MinMaxScaler. See Fig. 2a for the detailed descriptions of data loading and preprocessing in IOAT.

#### Feature selection

Multi-omics datasets are usually high-dimensional and contain noise, which necessarily requires feature dimension reduction. IOAT provides three analysis functions based on single factor analysis: (1) Correlation method, (2) Univariate Cox [4] regression method, and (3) Logrank test method. The tool also offers two analysis functions based on the multi-factor analysis method: (1) Multivariate Cox [4] regression, and (2) Lasso Cox model [5], as shown in Fig. 2a.

Users can directly use a single method or combine some of them. For the latter, where sequential feature screenings are performed, each feature screening will be performed on the remaining features after the previous feature screening. After each screening, a coefficient map of the relationship between features and a heat map of the sample features are drawn. Besides, a Lasso path map is given when the Lasso feature filtering is performed, which allows users to see the change of each regression coefficient with the penalty coefficient.

**Fig. 3:**
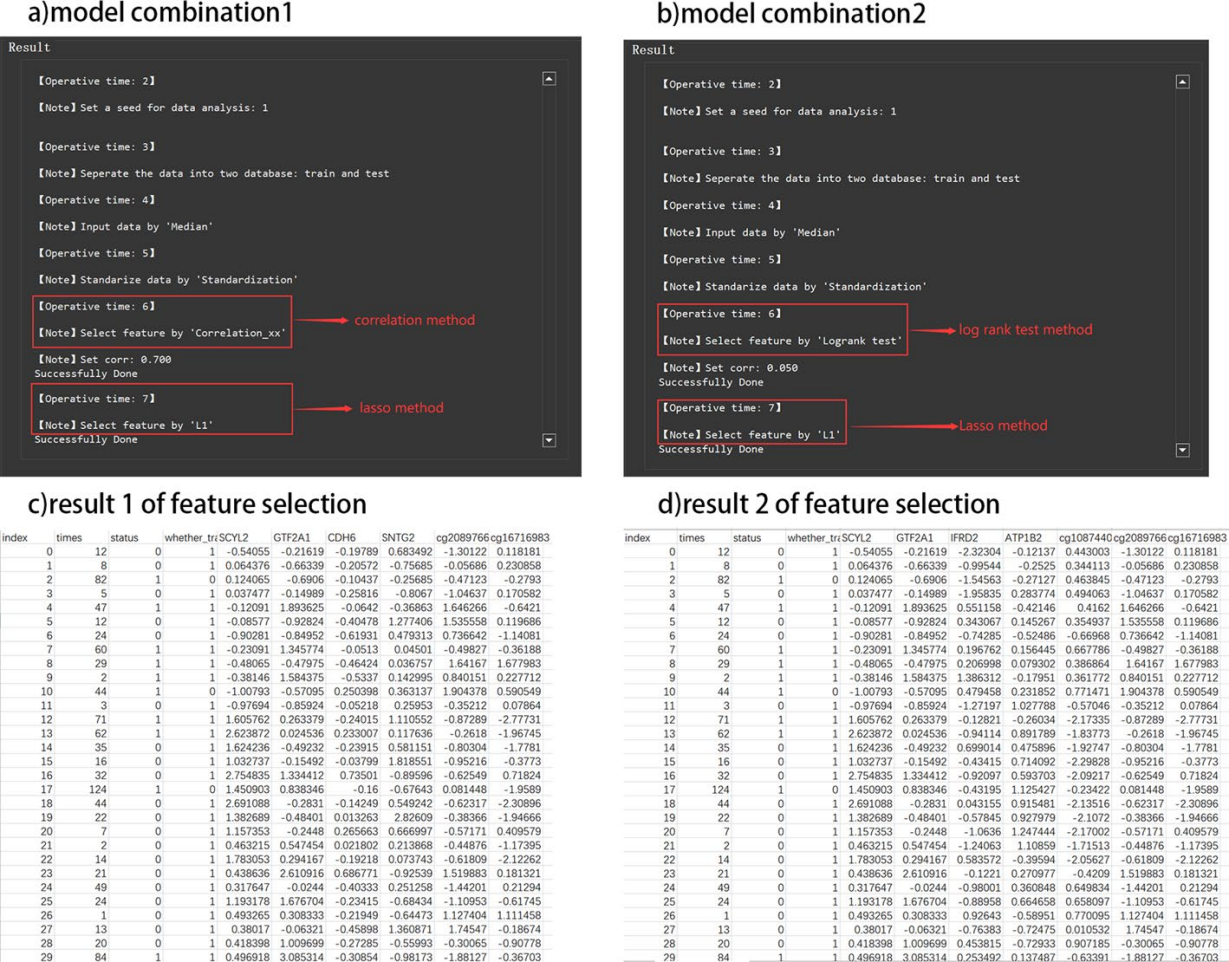
**a** The model combination 1 of feature selection setting. **b** The model combination 2 of feature selection setting. **c** The result 1 of feature selection. **d** The result 2 of feature selection

**Fig. 4:**
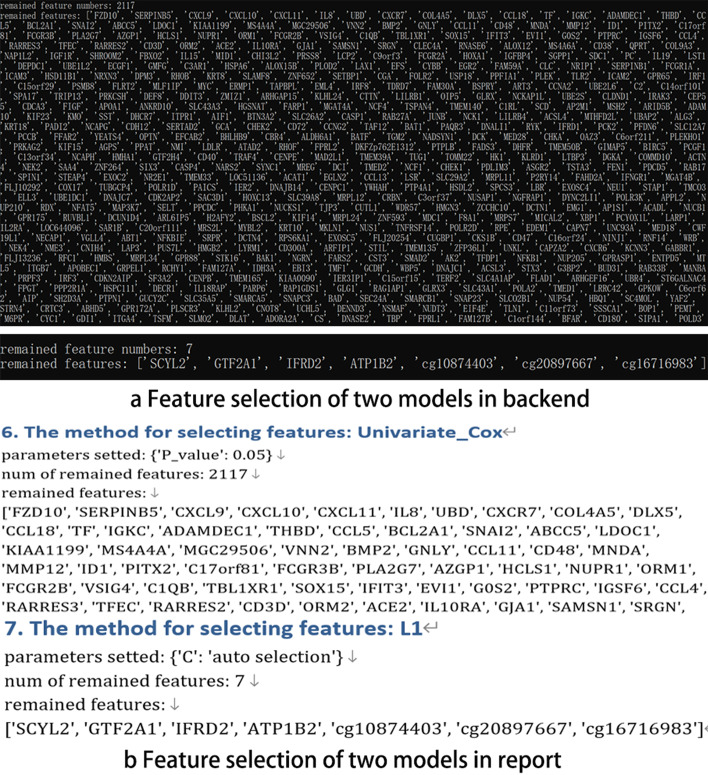
**a** The feature selection in the backend. **b** The feature selection in the report

Multi-omics (gene expression, methy expression, and mirna expression) data of lung cancer are taken as an example to illustrate the feature screening of the tool. We use single-factor and multi-factor methods in pairs, tested on a total of 7 combination models, as shown in Table 1, and select two models to compare the effect through risk assessment and survival analysis. According to the model combination of the saved six features, the first model we selected is to use the Correlation method (set 0.7 as its threshold) and Cox model (set 0.05 as its threshold). Although there are two combinations of models that retain the seven features, the results of the retained features are consistent, so the second model we selected is to use Log-rank test model (set 0.05 as its threshold) and the Lasso model. Specific steps and feature selection results of lung cancer can be retained at the user specified location (Fig. 3 (a) Operative time 6–7, (b) Operative time 6–7). Part of the visualization results are shown in Fig. 1 Step 2–3. Finally, we save the result of feature selection in the location we specify. The result of feature selection is shown in Fig. 3c, d. The results obtained based on the two sets of model feature selection results are: (1) the six features: ‘SCVL2’, ‘GTF2A1’, ‘IFRD2’, ‘ATP1B2’, ‘cg10874403’, ‘cg20897667’, ‘cg16716983’, and (2) the seven features are ‘SCVL2’, ‘GTF2A1’, ‘IFRD2’, ‘ATP1B2’, ‘cg10874403’, ‘cg20897667’, ‘cg16716983’. The result of feature selection can be found in the backend or output report (the report can be downloaded from https://github.com/WlSunshine/IOAT-software/blob/master/report.doc), as shown in Fig. 4a, b. In the result of feature selection, the user can manually select the feature of interest for research, and can select different features for different multi-omics data (genomics, clinical, etc.). Among them, most of characteristics are all derived from gene expression omics data, which shows that gene expression has a significant impact on the formation of lung cancer. Note that, the feature selection gives those genes related to tumors that may be connected to clinical phenotypes.

**Table 1:** Summary of method operation results

Method	Feature number after feature selection	Feature number after two feature selection
Correlation xx	10449	NULL
Cox model	2117	NULL
Log-rank test	16304	NULL
Lasso model	7	NULL
Correlation xx + Lasso	10449	6
Cox model + Lasso	2117	7
Log-rank test + Lasso	16304	7

#### Risk assessment

**Fig. 5:**
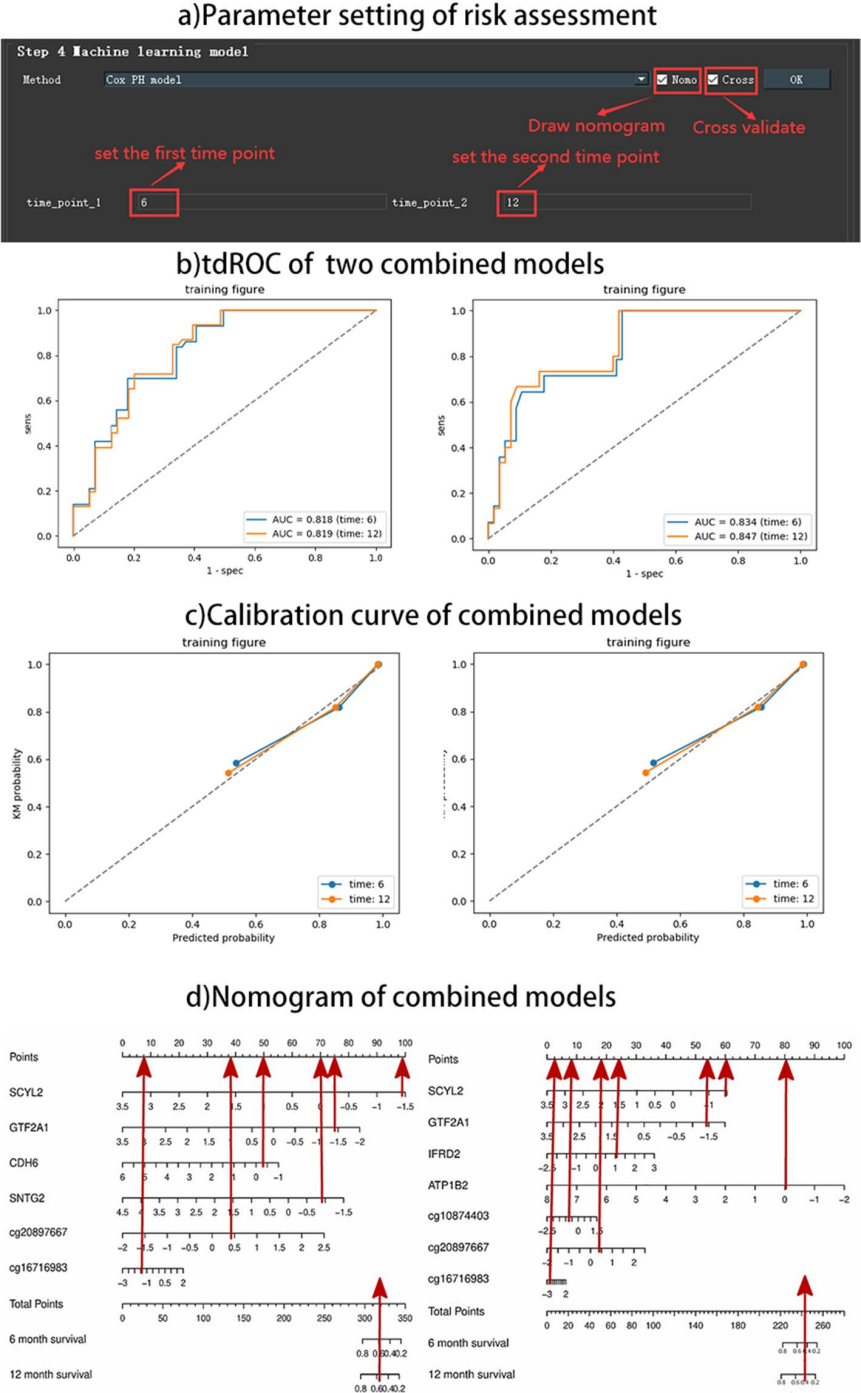
**a** Parameter setting of risk assessment. **b** tdROC of two combined model. **c** Calibration curve of two combined model. **d** Nomogram of two combined model

The Cox model risk assessment and prediction module provides the Cox model for users to choose. Users can use the multi-factor regression model Cox to calculate the characteristic risk value radscore (linear combination of regression coefficient $\beta$) and predict the probability of risk occurring in a certain period of time in the future. The system provides users with two choices: to draw a nomogram [6] or to perform cross-validation. When clicking the Nomo selection frame, the user can choose to set the forecast time (the default is 6 months in the future), as shown in Fig. 5a. Then click the OK button to perform the Cox model risk assessment and prediction, and draw a nomogram based on the risk assessment. Risk value radscore of each feature is assigned to each of their value levels. Then add the scores to get the total score. And finally use the function conversion relationship between the total score and the probability of the outcome even to calculate the predicted probability of the individual outcome event (such as the probability of cancer survive in the next 6 months). When the users click the Cross selection box, the model is trained by cross-validation, which makes the model more robust.

According to the results of feature selection of lung cancer multi-omics data in the last section, we assess the survival risk of the two groups (prediction time is 6 months and 12 months) of features respectively, and obtain their tdROC [7] diagram, calibration curve and nomogram, as shown in Fig. 5b–d. We compared their results and found that: the AUC obtained by the tdROC curve of the seven-featured model was 0.834 and 0.847, respectively, which were higher than the AUC 0.818 and 0.819 of the ROC of the six-featured model. On the calibration curve, the seven-feature model also performed slightly better than the six-feature model; you can see on the nomogram that the 6-month and 12-month survival rates predicted by the seven-feature model are 0.45, 0.4 respectively. That is lower than 0.6 and 0.58 of the 6-month and 12-month survival rates predicted by the six-characteristic model.

#### Clustering

**Fig. 6:**
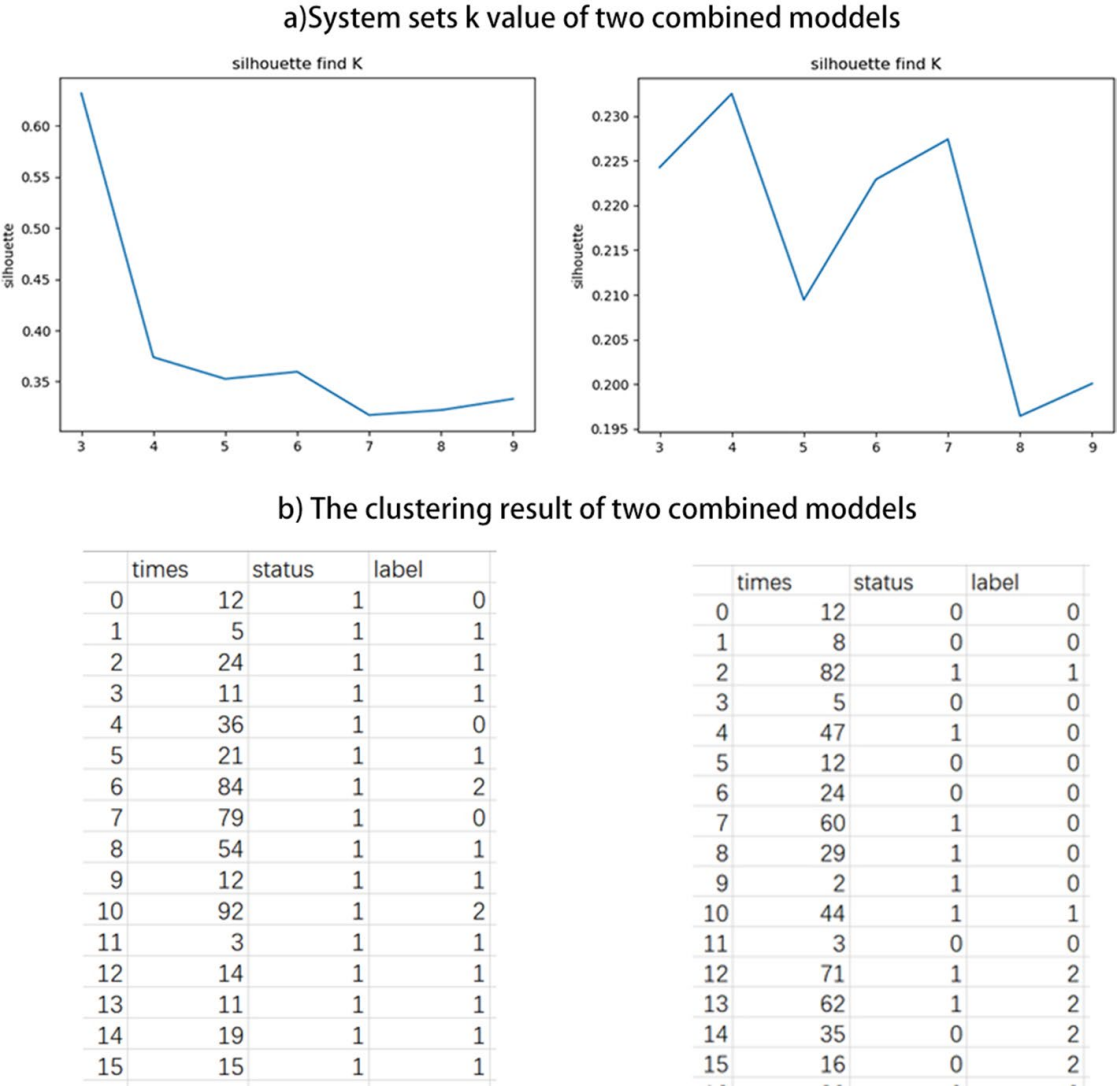
**a** Sysytem sets *k* value of two combined models. **b** The clustreing result of two combined models

Before performing the clustering operation, this module provides data preprocessing functions, including outlier elimination, filling missing values with either mean or median values, and feature scaling with either Standardization or MinMaxScaler.

Next, IOAT proposes different KMeans clustering methods for finding *k* values for two situations: (1) For labeled data (tumor comes with staging label), we use the AMI method to find the best cluster number which ranges from 3 to 9, and draws the graph of the correlation coefficient and the *k* value to discover new sub-categories to promote the development of precision medicine. (2) For unlabeled data, we use the silhouette method to find the best number of clusters, which also ranges from 3 to 9, and draws the graph of the correlation coefficient and the *k* value to classify the molecular subtypes of a particular cancer, as shown in Fig. 1 Step 5.

Besides, the user can also select the number of clusters (the default value is (3) by himself. The results obtained by clustering will be automatically saved on the user’s desktop for the next analysis,as shown in Fig. 2b.

According to the two model results obtained in the previous section, we performed KMeans clustering on the results of the selected six features and seven features respectively, and the results are shown in Fig. 6. Among them, (a) shows that the system finds the best *k* value for unlabeled lung cancer by the silhouette method. For the methods of Correlation and Lasso, the best clustering result obtained by the system is 4. For the methods of Cox model and Lasso, the best clustering result obtained by the system is 3, as shown in Fig. 6a, b. In order to better compare the results of the two models, we also manually set the number of clusters to 3 and 4 in order. (c) shows the csv result of clustering by the system with the best *k* value and by the user,as shown in Fig. 6b.

#### Survival analysis and visualization

**Fig. 7:**
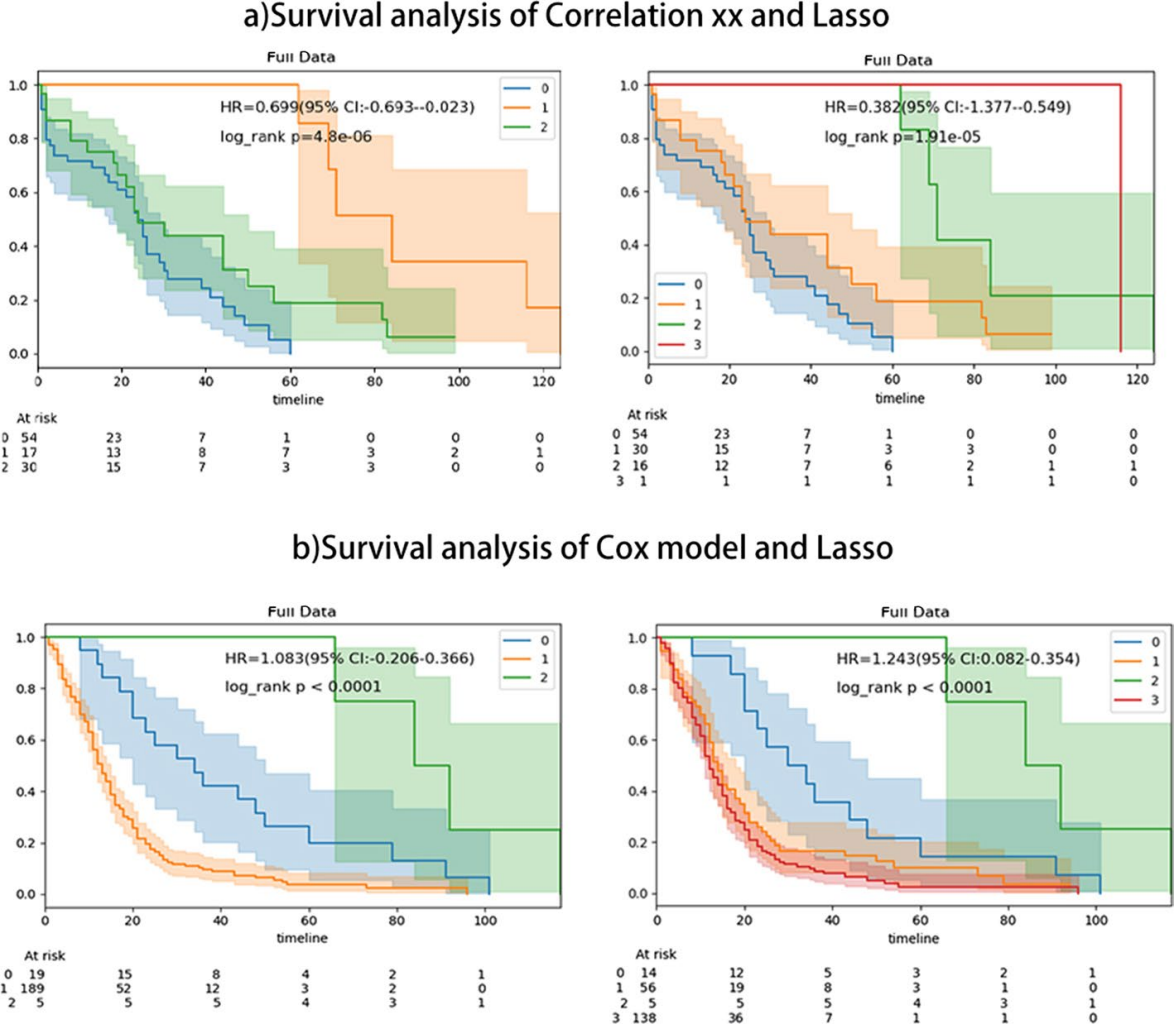
**a** Survival analysis of Correlation xx and Lasso. **b** Survival analysis of Cox model and lasso

In order to evaluate the clustering results obtained above, IOAT performs a survival analysis of the clustering results and displays a graph of the survival analysis result. Specifically, (1) survival analysis result on the *k* value selected by the user and the *k* value selected by the system are used to compare the similarities and differences between the choice of the user and the system. (2) A logrank test for each survival analysis chart is performed to verify whether there is a significant difference between different subtypes [8]. (3) The *HR* value is provided to show the ratio of the risk of incidents between different groups and the *CI* value is provided to show the confidence interval. (4) The survival timeline is drawn to show the number of survivors remaining in each time period [9], as shown in Fig. 2c. The specific process and related operation results of survival analysis taking lung cancer as an example are shown in Fig. 7. The result of survival analysis shows the *p* value of the methods of Cox model and Lasso is smaller than that of the methods of Correlation xx and Lasso, and the gap between each cluster is larger. Moreover, the effect in risk assessment is also better. So in summary, we can find that the methods of Cox model and Lasso are more suitable for the lung cancer data.

In addition, IOAT also provides the function of survival analysis of discrete features (such as male and female) in multi-omics data or clinical data.

## Discussion

Recent cancer projects such as TCGA [10], GDC [11], ICGC [12] as well as the GEO [13] database, provide the research community with a wealth of omic profiles and extensive clinical information on cancer patients [14]. And there are many multi-omics data analysis software, but they have some shortcomings.

Many tools have traditionally specialized in only one or perhaps a few data types. The IOAT tool we proposed can analyze multiple omics data of different integration types. In terms of data preprocessing and model selection, many multi-omics data analysis tools do not provide users with flexible choices. The IOAT tool can provide users with a variety of flexible choices, and can combine different models. In the future research, the tool will be developed into a new tool that can be compatible with users’ own methods to provide users with more choices. And many tools are provided to users to do multi-omics data research in the form of web-based. This method is very friendly to public data, but there is a risk of data leakage for users’ private data. The IOAT tool we propose is based on the user’s local use, which can guarantee the security of user private data. After downloading, users can analyze and train their own data without any network delay. After related tests, it is found that the tools UCSC Xena and Firehose do not completely combine multi-omics data with clinical data to carry out molecular subtype research. The IOAT tool we proposed mainly combines multi-omics data with clinical data to study molecular subtypes, and provides different clustering methods for unlabeled data and labeled data. In the investigation of the Firehose tool, we found that the tool outputs the results of cancer molecular subtypes into a fixed paper template. But our IOAT is to output the results of user data preprocessing, feature selection models and results, risk assessment results, clustering results, survival analysis results and visualization maps as a whole into a report, and provide it to users, thus clearly telling users every step of the operation, the setting of each parameter, and display the user’s usage time in the report.

IOAT aims to fill the gaps in the available analysis tools for such large genomic and clinical cancer data sets. At present, IOAT has been provided to users as a convenient installable executable file. In future, we will further enhance the tool to provide more feature selection methods and data preprocessing methods, such as adding this function of genomics and radiology to explore the association among them, deep learning framework, log2 transformation and upper-quartile normalization. In the follow-up, with the continuous function expansion of the software, for public data sets, we will launch a web-based multi-omics data analysis tool which provides functions like IOAT; For private data, We will expand the functions of the IOAT desktop software so that it can be compatible with the methods users want to use and add them by themselves.

## Conclusion

IOAT offers an easy-to-use and flexible tool for processing, per-forming dimensionality reduction, clustering, and visualizing multi-omics and clinical data. The tool’s one-click mouse service is convenient for non-technical users to perform research on private high-dimensional multi-omics data without security risks and any programming burden. The subtype classification results of a specific cancer can be easily obtained by the tool. At the same time, the tool accepts the combination of multiple multi-omics data, provides a variety of flexible data processing methods, and outputs the data processing process in the form of reports.

## Availability and requirements

- Project name: IOAT (An interactive tool for statistical analysis of omics data and clinical data).
- Project home page: https://github.com/WlSunshine/IOAT-software.
- Operating system: Windows
- Programming language: Python and R
- Other requirements: Installation of Python v3.5.6 or higher (for Windows), R v3.5.1 or higher (for Windows).
- License: GNU GPL 3.0
- Any restrictions to use by non-academics: None

## Data Availability

Software and data are available at https://github.com/WlSunshine/IOAT-software.

## Abbreviation

IOAT: An interactive tool for statistical analysis of omics data and clinical data
